# Facile Three-Component Synthesis, Insecticidal and Antifungal Evaluation of Novel Dihydropyridine Derivatives

**DOI:** 10.3390/molecules23102422

**Published:** 2018-09-21

**Authors:** Guan-Zhou Yang, Xiao-Fei Shang, Pi-Le Cheng, Xiao-Dan Yin, Jia-Kai Zhu, Ying-Qian Liu, Jing Zhang, Zhi-Jun Zhang

**Affiliations:** 1 School of Pharmacy, Lanzhou University, Lanzhou 730000, China; yanggzh2016@lzu.edu.cn (G.-Z.Y.); shangxiaofei@caas.cn (X.-F.S.); chengpile@hopelife.cn (P.-L.C.); yinxd14@lzu.edu.cn (X.-D.Y.); zhujk17@lzu.edu.cn (J.-K.Z.); 2 Lanzhou Institute of Husbandry and Pharmaceutical Sciences, Chinese Academy of Agricultural Sciences, Lanzhou 730000, China; 3 Environment and Plant Protection Institute, Chinese Academy of Tropical Agricultural Sciences, Haikou 571010, China

**Keywords:** neonicotinoid, insecticide, fungicide, dihydropyridine derivatives, three-component reaction

## Abstract

In an attempt to find the neonicotinoid insecticides, twenty novel dihydropyridine derivatives were designed, “green” synthesized via one pot facile three-component reaction and evaluated for their bioactivities against *Tetranychus cinnabarinus*, *Myzus persicae*, *Brevicoryne brassicae*, *Fusarium oxysporum* f. sp. *vasinfectum*, *Magnaporthe oryzae*, *Sclerotinia sclerotiorum* and *Botrytis cinereal*. All of the tested compounds showed potent insecticidal activity, and some were much better in comparison with imidacloprid (IMI). Especially, compounds **3d** (LC_50_: 0.011 mM) and **5c** (LC_50_: 0.025 mM) were 12.2- and 5.4-fold more active than IMI (LC_50_: 0.135 mM) against *T. cinnabarinus*, respectively. Moreover, out of all the derivatives, compound **3d** (LC_50_: 0.0015 mM) exhibited the strongest insecticidal activity against *B. brassicae* and compound **3i** (LC_50_: 0.0007 mM) displayed the strongest insecticidal activity against *M. persicae*. Surprisingly, when the concentration of compound **4** was 50 mg/L, the inhibition rate against *F. oxysporum* and *S. sclerotiorum* reached 45.00% and 65.83%, respectively. The present work indicated that novel dihydropyridine derivatives could be used as potential lead compounds for developing neonicotinoid insecticides and agricultural fungicides.

## 1. Introduction

Neonicotinoid insecticides are one of the most important chemical classes of insecticides introduced to global markets due to their broad spectrum of biological activities, favorable safety profile and unique mechanism of action [1], which have been registered globally in more than 120 countries for more than 25% of global insecticide market [2,3]. However, the great success of commercialization and the widespread and frequent use of these insecticides have inevitably led to the occurrence of resistance and cross-resistance [1,4,5,6]. In addition, reports of the toxicity of neonicotinoid insecticides on honey bees have raised concerns about whether the ecological balance has been destroyed [7,8]. Therefore, it is of great significance to seek a novel structure that is more efficient and less toxic as a potential candidate for future pest control.

**Figure 1:**
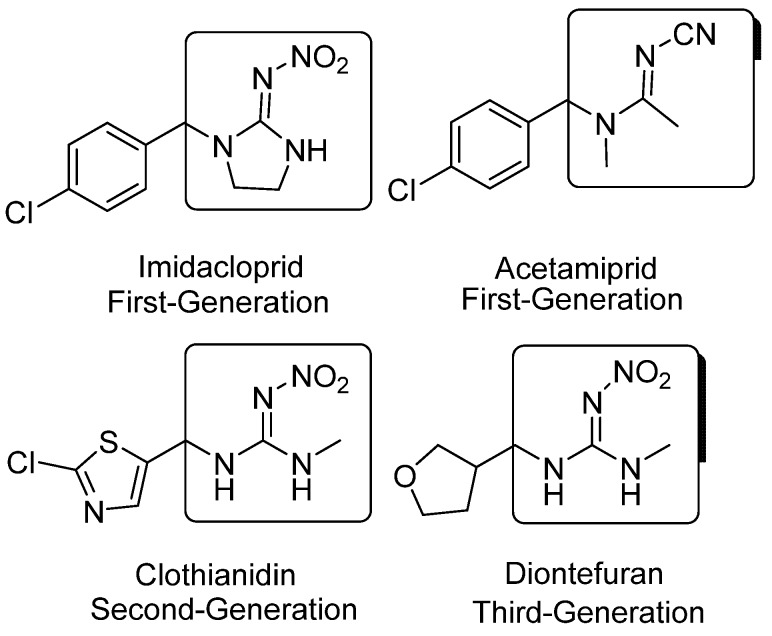
Representative generation of commercial neonicotinoid insecticides.

**Figure 2:**
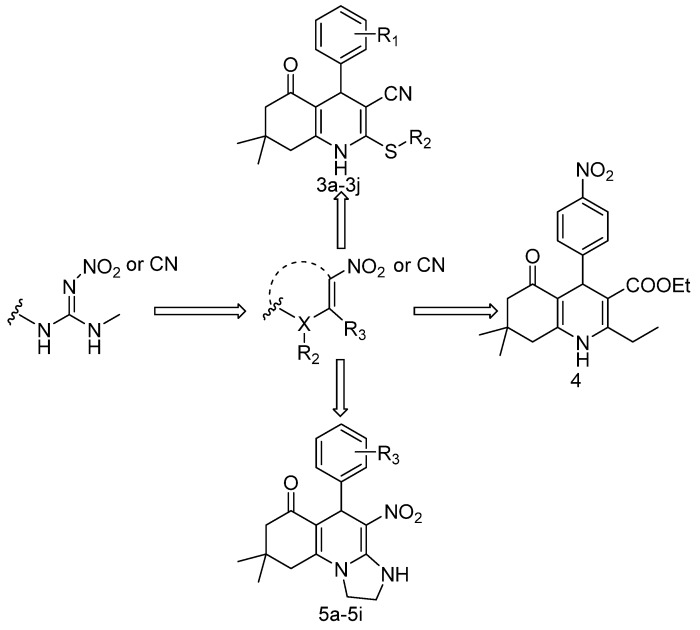
The molecular design of target compounds.

Representative generation of commercial neonicotinoid insecticides is shown in Figure 1. The common molecular structural features of neonicotinoids consist of four sections: (i) aromatic heterocycle, (ii) flexible linkage, (iii) hydroheterocyle or guanidine/amidine and (iv) electron-withdrawing segment [9,10] Through our previous work on pesticides [11,12,13,14] and other reported structure activity relationships for neonicotinoid insecticides [10], it has been found that pesticides containing dihydropyridine or dihydropyran rings have relatively low non-target organisms and environmental risks, high target specificity and a wide range of biological uses [15,16]. Furthermore, in recent years, multi-component reactions (MCRs) have become powerful tools for the synthesis of target molecules in organic chemistry, which are efficient, convenient, economical, practical and avoid purification and measurement of intermediate structures during the synthesis process [17,18]. On this basis, minimizing the amount of toxic waste and by-products and performing the reaction in the absence of non-environmental organic solvents is one of the goals that participated with green chemistry and green pesticides, as they were usually used in large quantities [19]. Thus, a series of derivatives with dihydropyridine as the core structure were designed and “green” synthesized by one-pot and three-component reactions and evaluated for their activity against *T. cinnabarinus*, *M. persicae*, *B. brassicae*, and four kinds of phytopathogens (Figure 2).

## 2. Results and Discussion

### 2.1. Chemistry

**Scheme 1:**
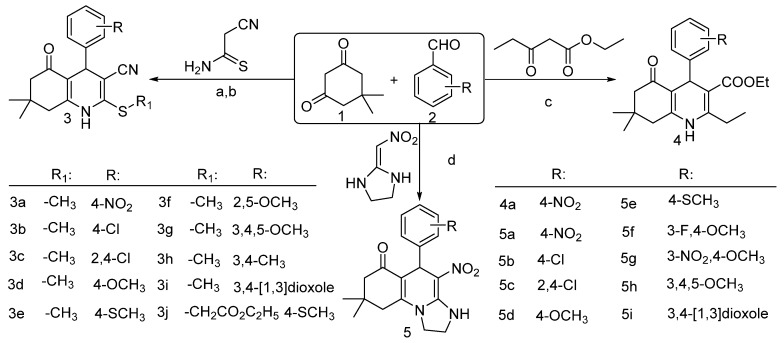
General synthetic procedure for target compounds **3a**–**3j**, **4** and **5a**–**5i**. Reagents and conditions: (**a**) Et_3_N, EtOH, reflux; (**b**) CH_3_I or CH_2_ICO_2_C_2_H_5_, EtOH, reflux; (**c**) HOAc, rt; (**d**) Et_3_N, EtOH, reflux.

MCRs provide an efficient, economical, and rapid approach towards the efficient synthesis of diverse compounds and compound libraries. The combination of MCR-type chemistry-planning with evolutionary in vitro prediction of drug potential or biological properties is a new and powerful tool in drug discovery [20]. The derivatives of **3a**–**3j**, **4a** and **5a**–**5i** were prepared according to the synthetic Scheme 1. Compounds **3a**–**3j** were produced by the three-component one-pot reaction of dimedone (1), aromatic aldehydes (2), and 2-cyanothioacetamide in the presence of morpholine under ethanol conditions at reflux temperature, and then further replaced by iodoalkane. Compound **4a** was produced by the one-pot three-component reaction of dimedone (1), aromatic aldehydes (2), and ethyl propionate under acetic acid conditions at room temperature. The reaction conditions of the **5a**–**5i** were substantially the same as those of **3a**–**3j** except that 2-cyanothioacetamide was replaced with and 2-(nitromethylene) imidazolidine. The structures of synthesized **3a**–**3j**, **4** and **5a**–**5i** were characterized by melting point, ^1^H-NMR, ^13^C-NMR and MS. The ^1^H and ^13^C NMR spectra of representative compounds can be found in the supplementary materials. The obtained results showed consistency with the expected structures and formulas of the targeted products. According to the reaction route, the reaction could occur under the conditions of ethanol and acetic acid as a solvent, in line with the importance of economical and green transformations in green chemistry.

### 2.2. Evaluation of Insecticidal Activity

Based on the methodology in Scheme 1, with twenty derivatives **3a**–**3j**, **4a** and **5a**–**5i** in hand, we examined their acaricidal activities against *T. cinnabarinus* and the results were summarized in Table 1. Next, we selected the five most active compounds (**3c**, **3d**, **3i**, **5c** and **5e**) to evaluate their insecticidal activities against *M. persicae* and *B. brassicae* and the results were shown in Table 2 and Table 3. IMI were tested under the same conditions as a comparison compound.

As indicated in Table 1, all new compounds exhibited potent acaricidal activity against *T. cinnabarinus*, with LC_50_ values ranging from 0.011 to 0.523 mM. Almost all of the derivatives showed significant acaricidal activities against *T. cinnabarinus*, and even half of the compounds were more than that of imidacloprid (0.135 mM). In particular, compounds **3c**, **3d**, **3i**, **5c** and **5e** showed pronounced acaricidal activities with respective LC_50_ values of 0.057, 0.011, 0.033, 0.025 and 0.064 mM, respectively, higher than that of imidacloprid (IMI) (0.135 mM). The activities of these compounds in Table 1 varied drastically, depending upon the types and patterns of substitution on the phenyl ring and dihydropyridinecore.

For the effect of substituents at phenyl ring in the series of **3a**–**3i** compounds, it was observed that compounds with electron-donating is favorable for high activities from data analysis present, the introduction of electron-donating groups, like **3d** (0.011 mM), **3e** (0.282 mM), **3f** (0.122 mM), **3g** (0.076 mM) and **3i** (0.033 mM), resulted in higher acaricidal potency than the corresponding analog with electron-withdrawing group, such as **3a** (0.473 mM) and **3b** (0.523 mM). Unexpectedly, the activity was found to increase rapidly when chlorine group was simultaneously introduced at the 2,4-position of the phenyl ring (**3c**, 0.057 mM), and its activity was nearly 10 times higher than that of the **3b** (0.523 mM), which introduced the chlorine group only at the 4-position the phenyl ring. Furthermore, the insecticidal activity of compound **3j** (0.079 mM) with an ethyl acetate group quadrupled compared to **3e** (0.282 mM) containing a methylthio group. In order to explore the effect of ester group on acaricidal activity, we replaced cyano group (**3a**, 0.473 mM) with ethyl formate (**4a**, 0.204 mM) and found that the activity was doubled. Of course, this may be due to the replacement of thiomethyl with ethyl. To identify more potent acaricidal activity, the effect of different phenyl groups on the insecticidal activity of the **5a**–**5j** derivatives was investigated. However, the structure activity relationship of **5a**–**5i** derivatives was completely different from that of **3a**–**3j**. Remarkably, compounds **5a**, **5c** and **5e** exhibited significant acaricidal activity against *T. cinnabarinus*, with LC_50_ values of 0.096, 0.025 and 0.064 mM, respectively. This indicated that the change in activity of the compound **5** series depended not only on the type of electronic effect of the substituent group on the benzene ring, but also on the number and position of the substituents. By summarizing the structure activity relationship of all compounds, the results clearly underlined that the acaricidal difference could be ascribed to combination of factors, like nature of the substitutes (which may depend on electronic characteristics of substitutes, the position of substitutes, and other factors) or by a different interaction at the site.

Subsequently, **3c**, **3d**, **3i**, **5c** and **5e** with the most acaricidal activities against *T. cinnabarinus* among of tested compounds, were selected to evaluate the insecticidal activity against *M. persicae* and *B. brassicae*. As to the insecticidal activities, from Table 2 and Table 3, it was surprising that all of the target compounds showed strong insecticidal activities against *M. persicae* and *B. oleracea*, and the activity of **3i** (0.0007 and 0.0025 mM) was even better than or equal to that of imidacloprid (0.0010 and 0.0006 mM). This result indicated that the series of compounds not only have good acaricidal activities, but also have extremely strong aphicidal activities. These encouraging results would prompt us to study the dihydropyridine derivatives as insecticidal agent in future.

### 2.3. Evaluation of Antifungal Activity

Plant fungal diseases are increasingly becoming a food security threat, and fungicides are widely used to control the development of phytopathogenic fungi. Inspired by the excellent insecticidal activities of this series of compounds, all compounds were evaluated for fungicidal activities against phytopathogenic fungi and it would inspire us to find a wider range of biologically active uses. Surprisingly, as shown in Table 4, although compound **4a** exhibited moderate insecticidal activity, it showed significant antifungal activity against *F. oxysporum* and *S. sclerotiorum* in vitro, and the inhibition rate reached 45.00% and 65.85% at 50 mg/L, respectively. The conversion of cyano group (**3a**) to ester groups (**4a**) could significantly increase the activities against phytopathogenic fungi, which provides a reference for our search to discovery the promising candidates with insecticidal and antifungal activities.

## 3. Experimental Section

### 3.1. Chemicals and Instruments

All reactions were performed with commercially available reagents without further purification. All reactions were monitored by thin-layer chromatography (TLC) and preparative thin-layer chromatography (PTLC) were performed with silica gel plates using silica gel 60 GF254 (Qingdao Haiyang Chemical Co., Ltd., Qingdao, China). Melting points were determined in an open capillary using WRS-2U melting point apparatus (Shanghai Precision Instrument Co., Ltd., Shanghai, China) and are uncorrected. Mass spectra were recorded on a Bruker Daltonics APEXII49e spectrometer (Bruker Daltonics Inc., Billerica, MA, US.) with ESI source as ionization. ^1^H and ^13^C-NMR spectra were recorded at 400 and 100 MHz on a Bruker AM-400 (Bruker Company, Billerica, MA, US.) spectrometer using TMS as reference. The commercial insecticide imidacloprid and fungicides azoxystrobin (analytical grade, 98% purity) (Jiangsu Bailing Agrochemical Co., Ltd., Jiangying, China) was used as a positive control in vitro experiment. *Tetranychus Cinnabarinus*, *Myzus persicae*, *Brevicoryne brassicae*, *Fusarium oxysporum* f. sp. *vasinfectum*, *Magnaporthe oryzae*, *Sclerotinia sclerotiorum* and *Botrytis cinereal* were obtained from the Institute of Plant Protection, Gansu Academy of Agricultural Science, Lanzhou, China.

### 3.2. Synthesis

#### 3.2.1. General Synthetic Procedure for Target Compounds **3a**–**3j**

A 0.5 L round-bottom flask fitted with an overhead stirrer was charged with the corresponding aromatic aldehyde (0.1 mol), cyanothioacetamide 10 (10.0 g, 0.10 mol) and EtOH (100 mL). Triethylamine (0.8−1.0 mL) was added, and the mixture was stirred for 1 h at 20 °C (yellow/orange crystalline may precipitate from the solution). Then dimedone (15.0 g, 0.104 mol) and *N*-methylmorpholine (16.5 mL, 0.15 mol) were added, and the solution was refluxed for 2−4 h, The mixture of salt was added iodomethane (0.1 mol) or ethyl iodoacetate (0.1 mol) in 80% ethanol (15 mL) and boiled for 2 min and then filtered through paper (Scheme 1). The solid was recrystallized from EtOH to afford the pure products [21].

**3a**: Yellow solid; yield 56%; m.p. 192–194 °C; ^1^H-NMR (400 MHz, DMSO-*d*_6_) δ: 9.75 (s, 1H, br, NH), 8.19 (d, 2H, *J* = 8 Hz, Ar-H), 7.44 (d, 2H, *J* = 8 Hz, Ar-H), 4.76 (s, 1H, CH), 2.54 (s, 3H, -SCH_3_), 2.42 (d, 2H, *J* = 16 Hz, CH_2_), 2.21 (d, 1H, *J* = 16 Hz, CH), 2.09–1.99 (m, 1H, CH), 1.03 (s, 3H, -CH_3_), 0.90 (s, 3H, -CH_3_). ^13^C-NMR (100 MHz, DMSO-*d*_6_) δ 194.1, 149.5, 148.3, 144.9, 144.4, 127.0, 127.0, 123.5, 123.5, 118.6, 111.9, 104.5, 51.2, 39.6, 32.8, 31.3, 29.7, 26.7, 17.1. HRMS: calcd. for C_19_H_19_N_3_O_3_S [2M + Na]: 761.2192, found: 761.2195.

**3b**: Yellow solid; yield 53%; m.p. 201–203 °C; ^1^H-NMR (400 MHz, DMSO-*d*_6_) δ: 9.66 (s, 1H, br, NH), 7.36 (d, 2H, *J* = 8 Hz, Ar-H), 7.16 (d, 2H, *J* = 8 Hz, Ar-H), 4.49 (s, 1H, -CH), 2.52 (s, 3H, SCH_3_), 2.43–2.46 (m, 2H, CH_2_), 2.20 (d, 1H, *J* = 16 Hz, CH), 2.01 (t, 1H, *J* = 16 Hz, CH), 1.02 (s, 3H, CH_3_), 0.89 (s, 3H, CH_3_). ^13^C-NMR (100 MHz, DMSO-*d*_6_) δ 194.6, 152.1, 145.5, 140.2, 131.3, 131.4, 131.4, 128.8, 128.8, 118.6, 111.9, 104.5, 51.2, 39.6, 32.8, 31.3, 29.7, 26.7, 17.1. HRMS: calcd. for C_19_H_19_ClN_2_OS [2M + Na]: 739.1711, found: 739.1720.

**3c**: Yellow solid; yield 48%; m.p. 243–245 °C; ^1^H-NMR (400 MHz, DMSO-*d*_6_) δ: 9.67 (s, 1H, br, NH), 7.54 (s, 1H, Ar-H), 7.39 (dd, 1H, *J* = 8 Hz, *J* = 4 Hz, Ar-H), 7.24 (d, 1H, *J* = 8 Hz, Ar-H), 4.99 (s, 1H, CH), 2.50 (s, 3H, -SCH_3_), 2.44–2.40 (m, 2H, CH _2_), 2.18 (d, 1H, *J* = 16 Hz, CH), 1.97–2.09 (m, 1H, CH), 1.03 (s, 3H, CH_3_), 0.93 (s, 3H, -CH_3_). ^13^C-NMR (100 MHz, DMSO-*d*_6_) δ 194.6, 152.1, 145.5, 140.2, 131.3, 131.4, 131.4, 129.5, 127.5, 118.6, 111.9, 104.5, 51.2, 39.6, 32.8, 31.3, 29.7, 26.7, 17.1. MS-ESI *m/z*: 807.0 [2M+Na] HRMS: calcd. for C_19_H_19_Cl_2_N_2_OS [2M + Na]: 807.0931, found: 807.0935.

**3d**: Yellow solid; yield 52%; m.p. 189–191 °C; ^1^H-NMR (400 MHz, DMSO-*d*_6_) δ: 9.59 (s, 1H, br, NH), 7.05 (d, 2H, *J* = 8 Hz, Ar-H), 6.85 (d, 2H, *J* = 8 Hz, Ar-H), 4.40 (s, 1H, CH), 3.71 (s, 3H, -OCH_3_), 2.50 (s, 3H, -SCH_3_), 2.38–2.45 (m, 2H, CH_2_), 2.19 (d, 1H, *J* = 16 Hz, CH), 1.99–2.09 (m, 1H, CH), 1.02 (s, 3H, -CH_3_), 0.90 (s, 3H, -CH_3_). ^13^C-NMR (100 MHz, DMSO-*d*_6_) δ 194.5, 157.8, 148.5, 146.2, 136.2, 133.3, 133.3, 118.6, 118.4, 118.4, 111.9, 104.5, 59.2, 52.7, 39.6, 32.8, 31.3, 29.7, 26.7, 17.1. MS-ESI *m/z*: 731.1 [2M + Na] HRMS: calcd. for C_20_H_22_N_2_O_2_S [2M + Na]:731.2702, found: 731.2710.

**3e**: Yellow solid; yield 55%; m.p. 197–199 °C; ^1^H-NMR (400 MHz, DMSO-*d*_6_) δ: 9.63 (s, 1H, br, NH), 7.18 (d, 1H, *J* = 12 Hz, Ar-H), 7.08 (t, 2H, *J* = 8 Hz, Ar-H), 4.42 (s, 1H, CH), 2.44 (m, 5H, CH_2_, SCH_3_), 2.50 (s, 3H, SCH_3_), 2.20 (d, 1H, *J* = 16 Hz, CH), 2.09–2.00 (m, 1H, CH), 1.03 (s, 3H, CH_3_), 0.90 (s, 3H, CH_3_). ^13^C-NMR (100 MHz, DMSO-*d*_6_) δ 194.5, 148.5, 146.2, 142.8, 140.3, 130.3, 130.3, 128.9, 128.9, 118.6, 111.9, 104.5, 52.7, 39.6, 32.8, 31.3, 29.7, 26.7, 17.1, 14.8. MS-ESI *m/z*: 763.0 [2M + Na] HRMS: calcd. for C_20_H_22_N_2_OS_2_ [2M + Na]: 763.2245, found: 763.2251.

**3f**: Yellow solid; yield 48%; m.p. 195–199 °C; ^1^H-NMR (400 MHz, DMSO-*d*_6_) δ: 9.54 (s, 1H, br, NH), 6.89 (d, 1H, *J* = 8 Hz, Ar-H), 6.73 (dd, 1H, *J* = 8 Hz, *J* = 4 Hz, Ar-H), 6.52 (d, 1H, *J* = 4 Hz, Ar-H), 4.80 (s, 1H, CH), 3.73 (s, 3H, OCH_3_), 3.64 (s, 3H, OCH_3_), 2.46 (s, 3H, -SCH_3_), 1.04 (s, 3H, CH_3_), 2.44–2.40 (m, 2H, CH_2_), 2.19 (d, 1H, *J* = 16 Hz, CH), 2.09–1.98 (m, 1H, CH), 0.96 (s, 3H, CH_3_). ^13^C-NMR (100 MHz, DMSO-*d*_6_) δ 194.5, 152.8, 151.2, 150.5, 146.2, 123.3, 118.6, 116.5, 112.8, 111.9, 106.5, 56.6, 55.9, 52.7, 39.6, 32.8, 29.7, 26.7, 25.3 17.1. MS-ESI *m/z*: 792.3 [2M + Na] HRMS: calcd. for C_21_H_24_N_2_O_3_S [2M + Na]: 791.2913, found: 791.2918.

**3g**: Light yellow solid; yield 55%; m.p. 221–223 °C; ^1^H-NMR (400 MHz, DMSO-*d*_6_) δ: 9.66 (s, 1H, br, NH), 6.40 (s, 1H, Ar-H), 4.42 (s, 1H, CH), 3.71 (s, 6H, OCH_3_), 3.67 (s, 3H, OCH_3_), 2.50 (s, 3H, SCH_3_) 2.45–2.41 (m, 2H, CH_2_), 2.23 (d, 1H, *J* = 16 Hz, CH), 2.09–1.99 (m, 1H, CH), 1.05 (s, 3H, CH_3_), 0.98 (s, 3H, CH_3_). ^13^C-NMR (100 MHz, DMSO-*d*_6_) δ 194.5, 152.8, 152.8, 150.5, 138.5, 137.2, 118.6, 111.9, 106.5, 106.5, 104.5, 61.6, 56.8, 56.8, 52.7, 39.6, 32.8, 31.9, 29.7, 26.7, 25.3 17.1. HRMS: calcd. for C_22_H_26_N_2_O_4_S [2M + Na]: 851.3124, found: 851.3129.

**3h**: Light yellow solid; yield 58%; m.p. 201–203 °C; ^1^H-NMR (400 MHz, DMSO-*d*_6_) δ: 9.77 (s, 1H, br, NH), 7.18 (d, 1H, *J* = 12 Hz, Ar-H), 7.08 (t, 2H, *J* = 8 Hz, Ar-H), 4.43 (s, 1H, CH) 4.04 (q, 2H, *J* = 8 Hz, CH_2_), 3.84 (dd, 2H, *J* = 16 Hz, *J* = 44 Hz, CH_2_), 2.37–2.41 (m, 5H, CH_2_, SCH_3_), 2.22–2.18 (d, 1H, *J* = 16 Hz, CH), 2.00–2.09 (m, 1H, CH), 1.13 (t, 3H, *J* = 4 Hz, CH_3_), 1.02 (s, 3H, CH_3_), 0.91 (s, 3H, CH_3_). ^13^C-NMR (100 MHz, DMSO-*d*_6_) δ 194.5, 150.5, 146.2, 140.2, 137.7, 134.2, 131.2, 130.8, 127.6, 118.6, 111.9, 106.5, 54.6, 39.6, 32.8, 31.6, 29.7, 26.7, 19.1, 18.8, 17.1. HRMS: calcd. for C_21_H_24_N_2_OS [2M + Na]: 727.3116, found: 727.3120.

**3i**: Light yellow solid; yield 51%; m.p. 195–197 °C; ^1^H-NMR (400 MHz, DMSO-*d*_6_) δ: 9.60 (s, 1H, br, NH), 6.82 (d, 1H, *J* = 8 Hz, Ar-H), 6.06 (t, 2H, *J* = 8 Hz, Ar-H), 5.07 (s, 2H, CH_2_), 4.39 (s, 1H, CH), 2.50 (s, 3H, -SCH_3_), 2.44 (d, 2H, *J* = 4 Hz, CH_2_), 2.19 (d, 1H, *J* = 16 Hz, CH), 2.02–2.09 (m, 1H, CH) 1.02 (s, 3H, CH_3_), 0.91 (s, 3H, CH_3_). ^13^C-NMR (100 MHz, DMSO-*d*_6_) δ 194.5, 150.5, 148.7, 145.8, 144.4, 135.5, 122.6, 118.6, 115.9, 112.3, 111.9, 106.5, 101.5, 54.6, 39.6, 32.8, 31.6, 29.7, 26.7, 17.1. HRMS: calcd. for C_20_H_20_N_2_O_3_S [2M + Na]: 759.2287, found: 759.2290.

**3j**: Yellow solid; yield 57%; m.p. 213–215 °C; ^1^H-NMR (400 MHz, DMSO*-d*_6_) δ: 9.57 (s, 1H, br, NH), 7.04 (d, 1H, *J* = 8 Hz, Ar-H), 6.84 (m, 2H, Ar-H), 4.36 (s, 1H, CH), 2.50 (s, 3H, SCH_3_), 2.43–2.46 (m, 2H, CH_2_), 2.17–2.15 (m, 6H, CH_3_), 2.09–1.99 (m, 2H, CH_2_), 1.03 (s, 3H, CH_3_), 0.91 (s, 3H, CH_3_). ^13^C-NMR (100 MHz, DMSO-*d*_6_) δ 194.5, 169.9, 150.5, 145.8, 139.8, 139.2, 130.5, 130.5, 128.6, 128.6, 118.6, 111.9, 106.5, 62.8, 54.6, 39.6, 34.2, 32.8, 31.6, 29.7, 26.7, 14.8, 14.1. HRMS: calcd. for C_23_H_26_N_2_O_3_S_2_ [2M + Na]: 907.2667, found: 907.2672.

#### 3.2.2. General Synthetic Procedure for Target Compounds **4**

In a dry 50 mL flask, arylaldehyde (1 mmol), ethyl propionylacetate (1 mmol), dimedone (1 mmol) and excessive ammonium acetate and HOAc (10 mL) were mixed and then stirred at room temperature for 8–10 h. After completion of the reaction, as indicated by thin layer chromatography (TLC), the reaction mixture was poured into water, then the solid product was collected and purified by flash column chromatography (Scheme 1) [22].

**4**: Yellow solid; yield 56%; m.p. 223–225 °C; ^1^H-NMR (400 MHz, DMSO*-d*_6_) δ: 9.22 (s, 1H, br, NH), 8.09 (d, 2H, *J* = 8 Hz, Ar-H), 7.40 (d, 2H, *J* = 8 Hz, Ar-H), 4.97 (s, 1H, CH), 3.94 (q, 2H, *J* = 8 Hz, CH_2_), 2.76–2.67 (m, 2H, CH_2_), 2.32–2.18 (m, 2H, CH_2_), 2.09–1.95 (m, 2H, CH_2_), 1.13–1.09 (m, 6H, CH_3_), 1.01 (s, 3H, CH_3_), 0.80 (s, 3H, CH_3_). ^13^C-NMR (100 MHz, DMSO-*d*_6_) δ 194.5, 169.9, 152.6, 150.5, 149.5, 145.8, 127.8, 127.8, 124.6, 124.6, 111.9, 102.5, 62.8, 54.6, 42.4, 40.3, 32.8, 29.7, 26.7, 23.8, 14.2, 12.9. HRMS: calcd. for C_22_H_26_N_2_O_5_ [2M + Na]: 819.3581, found: 819.3583.

#### 3.2.3. General Synthetic Procedure for Target Compounds **5a**–**5i**

2-(nitromethylene) imidazolidine (0.5 mmol), aldehydes (0.5 mmol), dimedone (0.5 mmol) and EtOH (10 mL) and Et_3_N (0.25 mmol) were added into a 25 mL flask and the mixture was stirred for the appropriate reaction time at 80 °C in an oil bath until the 2-(nitromethylene) imidazolidine was completely consumed. The solid mixture was washed with EtOH (2 × 5 mL). The crude residue was recrystallized from EtOH to afford the pure products (Scheme 1) [23].

**5a**: Dark yellow solid; yield 56%; m.p. 264–267 °C; ^1^H-NMR (400 MHz, DMSO-*d*_6_) δ 9.52 (s, 1H, br, NH), 8.14–7.97 (m, 2H, Ar-H), 7.63–7.42 (m, 2H, Ar-H), 5.16 (s, 1H, CH), 4.26–4.14 (m, 1H, CH), 4.05 (q, *J* = 9.5 Hz, 1H, CH), 3.91–3.77 (m, 2H, CH_2_), 2.62 (d, *J* = 17.7 Hz, 1H, CH_2_), 2.55 (s, 1H, CH_2_), 2.21 (d, *J* = 16.1 Hz, 1H, CH_2_), 2.00 (d, *J* = 16.1 Hz, 1H, CH_2_), 1.05 (s, 3H, CH_3_), 0.84 (s, 3H, CH_3_). ^13^C-NMR (100 MHz, DMSO-*d*_6_) δ 194.7, 160.8, 148.3, 146.5, 144.9, 134.2, 131.5, 128.89, 126.7, 111.9, 94.0, 52.2, 50.5, 39.9, 40.9, 33.1, 30.1, 29.7, 26.7. HRMS: calcd. for C_19_H_20_N_4_O_5_ [M + H]^+^: 385.1512, found: 385.1518.

**5b**: Yellow solid; yield 56%; m.p. 295–296 °C; ^1^H-NMR (400 MHz, DMSO-*d*_6_) δ 9.45 (s, 1H, br, NH), 7.34–7.14 (m, 4H, Ar-H), 5.50 (s, 1H, CH), 4.17 (dd, *J* = 10.0, 6.7 Hz, 1H, CH), 4.03(q, *J* = 9.5 Hz, 1H, CH_2_), 3.87–3.78 (m, 2H, CH_2_), 2.65 (d, *J* = 20.0 Hz, 1H, CH_2_), 2.58 (s, 1H, CH_2_), 2.20 (d, *J* = 16.1 Hz, 1H, CH_2_), 2.00 (d, *J* = 16.3 Hz, 1H, CH_2_), 1.05 (s, 3H, CH_3_), 0.85 (s, 3H, CH_3_). ^13^C-NMR (100 MHz, DMSO-*d*_6_) δ 193.7, 151.9, 150.0, 140.5, 134.3, 134.2, 131.5, 128.89, 126.7, 112.1, 106.7, 45.2, 43.9, 39.9, 38.8, 37.4, 32.1, 29.7, 26.7. HRMS: calcd. for C_19_H_20_ClN_3_O_3_ [M + Na]^+^: 396.1091, found: 396.1095.

**5c**: Yellow solid; yield 56%; m.p. 284–287 °C; ^1^H-NMR (400 MHz, DMSO-*d*_6_) δ 9.47 (s, 1H, br, NH), 7.48–7.33 (m, 2H, Ar-H), 7.26 (dd, *J* = 8.4, 2.2 Hz, 1H, Ar-H), 5.28 (s, 1H, CH), 4.19 (t, *J* = 9.0 Hz, 1H, CH), 4.08 (s, 1H, CH_2_), 3.84 (s, 2H, CH_2_), 2.60 (d, *J* = 17.6 Hz, 1H, CH_2_), 2.46 (s, 1H, CH_2_), 2.18 (d, *J* = 16.1 Hz, 1H, CH_2_), 1.98 (s, 1H, CH_2_), 1.05 (s, 3H, CH_3_), 0.87 (s, 3H, CH_3_). ^13^C-NMR (100 MHz, DMSO-*d*_6_) δ 193.7, 151.9, 150.0, 140.5, 134.3, 134.2, 131.5, 128.89, 126.7, 112.1, 106.7, 45.2, 43.9, 39.9, 38.8, 37.4, 32.1, 29.7, 26.7. HRMS: calcd. for C_19_H_19_Cl_2_N_3_O_3_ [M + Na]^+^: 430.0701, found: 430.0731.

**5d**: Yellow solid; yield 56%; m.p. 310–311 °C; ^1^H-NMR (400 MHz, DMSO-*d*_6_) δ 9.38 (s, 1H, br, NH), 7.22–7.05 (m, 2H, Ar-H), 6.86–6.63 (m, 2H, Ar-H), 5.02 (s, 1H, CH), 4.17 (s, 1H, CH_2_), 4.02 (d, *J* = 9.6 Hz, 1H, CH_2_), 3.83 (t, *J* = 9.9 Hz, 2H, CH_2_), 3.68 (s, 3H, CH_3_), 2.65 (d, *J* = 21.3 Hz, 1H, CH_2_), 2.58 (s, 1H, CH_2_), 2.19 (d, *J* = 16.1 Hz, 1H, CH_2_), 1.99 (d, *J* = 16.1 Hz, 1H, CH_2_), 1.05 (s, 3H, CH_3_), 0.86(s, 3H, CH_3_). ^13^C-NMR (100 MHz, DMSO-*d*_6_) δ 198.5, 162.7, 156.8, 153.9, 141.7, 134.2, 134.0, 119.1, 119.1, 118.2, 94.0, 60.1, 54.7, 50.0, 48.6, 43.4, 41.4, 37.0, 34.6, 31.4. HRMS: calcd. for C_20_H_23_N_3_O_4_ [M + H]^+^: 370.1767, found: 370.1771.

**5e**: Yellow solid; yield 56%; m.p. 279–281 °C; ^1^H-NMR (400 MHz, DMSO-*d*_6_) δ 9.42 (s, 1H, br, NH), 7.19–7.13 (m, 2H, Ar-H), 7.09 (d, *J* = 8.3 Hz, 2H, Ar-H), 5.03 (s, 1H, CH), 4.19 (q, *J* = 8.7 Hz, 1H, CH_2_), 4.03 (q, *J* = 9.6 Hz, 1H, CH_2_), 3.84 (d, *J* = 9.9 Hz, 2H, CH_2_), 2.58 (d, *J* = 17.0 Hz, 1H, CH_2_), 2.53 (s, 1H, CH_2_), 2.41 (s, 3H, CH_3_), 2.20 (d, *J* = 16.0 Hz, 1H, CH_2_), 2.00 (d, *J* = 16.1 Hz, 1H, CH_2_), 1.05 (s, 3H, CH_3_), 0.86 (s, 3H, CH_3_). ^13^C-NMR (100 MHz, DMSO-*d*_6_) δ 193.7, 152.1, 149.7, 141.6, 135.8, 128.9, 128.9, 126.0, 126.0, 114.0, 107.7, 49.9, 45.3, 43.9, 38.7, 37.2, 32.3, 29.8, 26.6, 15.3. HRMS: calcd. for C_20_H_24_N_3_O_3_S [M + H]^+^: 386.1538, found: 386.1540.

**5f**: Yellow solid; yield 56%; m.p. 278–279 °C; ^1^H-NMR (400 MHz, DMSO-*d*_6_) δ 9.42 (s, 1H, br, NH), 6.97 (d, *J* = 5.2 Hz, 3H, Ar-H), 5.02 (s, 1H, CH), 4.25–4.16 (m, 1H, CH_2_), 4.02 (q, *J* = 9.6 Hz, 1H, CH_2_), 3.83 (t, *J* = 8.6 Hz, 2H, CH_2_), 3.77 (s, 3H, CH_3_), 2.60 (d, *J* = 17.7 Hz, 1H, CH_2_), 2.54 (s, 1H, CH_2_), 2.18 (s, 1H, CH_2_), 2.03 (s, 1H, CH_2_), 1.05 (s, 3H, CH_3_), 0.87 (s, 3H, CH_3_). ^13^C-NMR (100 MHz, DMSO-*d*_6_) δ 193.8, 152.0, 149.5, 145.7, 124.3, 124.2, 115.7, 115.5, 113.7, 113.4, 107.5, 56.4, 45.3, 43.9, 40.5, 38.7, 36.9, 32.3, 29.8, 26.6. HRMS: calcd. for C_20_H_22_FN_3_O_4_ [M + H]^+^: 388.1673, found: 388.1675.

**5g**: Yellow solid; yield 56%; m.p. 279–280 °C; ^1^H-NMR (400 MHz, DMSO-*d*_6_) δ 9.47 (s, 1H, br, NH), 7.62 (d, *J* = 2.3 Hz, 1H, Ar-H), 7.51 (dd, *J* = 8.7, 2.3 Hz, 1H, Ar-H), 7.19 (d, *J* = 8.7 Hz, 1H, Ar-H), 5.04 (s, 1H, CH), 4.19 (td, *J* = 9.5, 6.9 Hz, 1H, CH), 4.08–3.94 (m, 1H, CH_2_), 3.85 (d, *J* = 10.8 Hz, 5H, CH_3_, CH_2_), 2.61 (d, *J* = 17.7 Hz, 1H, CH_2_), 2.55 (s, 1H, CH_2_), 2.21 (d, *J* = 16.1 Hz, 2H, CH_2_), 2.01 (d, *J* = 16.1 Hz, 2H, CH_2_), 1.06 (s, 3H, CH_3_), 0.88 (s, 3H, CH_3_). ^13^C-NMR (100 MHz, DMSO-*d*_6_) δ 193.8, 151.8, 150.8, 150.0, 139.1, 137.3, 134.4, 124.1, 113.9, 113.0, 107.2, 57.0, 49.8, 45.3, 43.9, 38.7, 37.2, 32.3, 29.8, 26.6. HRMS: calcd. for C_20_H_22_N_4_O_6_ [M + H]^+^: 415.1618, found: 415.1621.

**5h**: Yellow solid; yield 56%; m.p. 240–242 °C; ^1^H-NMR (400 MHz, DMSO-*d*_6_) δ 9.40 (s, 1H, br, NH), 6.46 (s, 2H, Ar-H), 5.09 (s, 1H, CH), 4.28–4.13 (m, 1H, CH), 4.02(q, *J* = 9.6 Hz, 1H, CH), 3.83 (dd, *J* = 10.2, 7.2 Hz, 2H, CH_2_), 3.70 (s, 6H, CH_3_, CH_3_), 3.60 (s, 3H, CH_3_), 2.63 (d, *J* = 17.7 Hz, 1H, CH_2_), 2.58 (s, 1H, CH_2_), 2.23 (d, *J* = 16.2 Hz, 1H, CH_2_), 2.05(d, *J* = 16.1 Hz, 1H, CH_2_), 1.07 (s, 3H, CH_3_), 0.94 (s, 3H, CH_3_). ^13^C-NMR (100 MHz, DMSO-*d*_6_) δ 198.6, 157.5, 156.8, 154.6, 157.5, 144.9, 141.3, 118.6, 112.3, 110.4, 110.4, 94.0, 65.1, 61.0, 61.0, 54.7, 48.6, 43.5, 42.0, 37.0, 34.8, 31.2. HRMS: calcd. for C_22_H_27_N_3_O_6_ [M + H]^+^: 430.1978, found: 430.1985.

**5i**: Yellow solid; yield 56%; m.p. 345–356 °C; ^1^H-NMR (400 MHz, DMSO-*d*_6_) δ 9.13 (s, 1H, br, NH), 6.74–6.65 (m, 3H, Ar-H), 5.91 (d, *J* = 3.8 Hz, 2H, CH_2_), 4.25 (d, *J* = 10.8 Hz, 1H, CH), 3.75–3.57 (m, 4H, CH_2_), 2.59 (d, *J* = 17.4 Hz, 1H, CH_2_), 2.44 (s, 1H, CH_2_), 2.11 (d, 1H, CH_2_), 1.92 (d, *J* = 14.2 Hz, 1H, CH_2_), 1.04 (s, 3H, CH_3_), 1.01 (s, 3H, CH_3_). ^13^C-NMR (100 MHz, DMSO-*d*_6_) δ 204.8, 157.6, 146.7, 145.3, 139.1, 135.5, 121.4, 109.4, 108.6, 107.8, 100.9, 85.6, 54.3, 46.3, 43.3, 36.6, 35.3, 33.3, 32.3, 28.1. HRMS: calcd. for C_20_H_23_N_3_O_6_ [M + H]^+^: 384.1559, found: 384.1565.

### 3.3. Insecticidal Activity

Bioassays on representative test organisms reared in the laboratory were carried out at 25 ± 2 °C apropos to statistical standards. Series concentrations of 250.0, 100.0, 50.0, 10.0, 1.0 and 0.1 mg/L for bioassays were obtained by dissolving all the synthesized dihydropyridine analogues in acetone and diluted with water containing Tween-20 (0.1 mg/L). The control imidacloprid was tested under the same experimental conditions.

#### 3.3.1. Acaricidal Assay against *T. Cinnabarinus*.

Slide immersion method recommended by FAO [13] was employed to evaluate the acaricidal activity of all the synthesized agents. All the test compounds were prepared in acetone at a concentration of 250 mg/L and diluted to the required concentration with distilled water containing TW-80. Using a small brush, thirty adult spider mites were fixed dorsally to a strip of double-sided tape attached to the slide. The slide was immersed diluted solution of the test compounds and shaken for 3 s. The treated slides with the mites were kept at 25 ± 2 °C in a covered dish with wet filter paper after the excessive solution was removed. After 24 h treatment, the number of demised mites was recorded. Each treatment was repeated with triplicate experiments and each replicate involved 30 adult mites. Control groups were tested with only acetone.

#### 3.3.2. Insecticidal Assay Against *M. Persicae* and *B. Brassicae*.

The insecticidal activities of five compounds **3c**, **3d**, **3i**, **5c**, **5e** and imidacloprid against *M. persicae* and *B. brassicae* were evaluated according to the reported procedure [24].

### 3.4. Antifungal Activity

The effects of **3a**–**j**, **4a**, **5a**–**i** and azoxystrobin on the mycelial growth against *F. oxysporum*, *M. oryzae*, *S. sclerotiorum* and *B. cinerea* were assessed using Poison Food Technique in solid media [25]. After completely covered the Petri dishes of the fungal, the mycelial growth diameters were measured and inhibition percentages relative to the control with DMSO were calculated using the formula from Agarwal: I(%) = ([(C − d) − (T − d)])/((C − d)) × 100, where d is diameter of the cut fungus (5 mm), I is the inhibition (%), and C and T are the average colony diameters of the mycelium of the control and treatment, respectively.

## 4. Conclusions

In summary, a series of novel dihydropyridine derivatives were designed, “green” synthesized via one pot facile three-component reaction and their structures were characterized by melting point, ^1^H-NMR, ^13^C-NMR and EI-MS. The bioactivities were evaluated against *T. cinnabarinus*, *M. persicae*, *B. brassicae*, *F. oxysporum*, *M. oryzae*, *S. sclerotiorum* and *B. cinereal*. In particular, Compound **3d** (LC_50_: 0.011 and 0.0015 mM) exhibited the strongest insecticidal activity against *T. cinnabarinus* and *B. brassicae* in all of the derivatives we prepared. Compound **3i** (LC_50_: 0.0007 mM) exhibited the strongest insecticidal activity against *M. persicae*, and, surprisingly, when the concentration of compound **4a** was 50 mg/L, the inhibition rate against *F. oxysporum* and *S. sclerotiorum* reached 45.00% and 65.83%. SARs clearly indicated that variations of R groups in the position of benzene ring markedly affected the insecticidal activity. When cyano group was replaced by ethyl acetate at the 3-position of dihydropyridine core, the biological activity spectrum was markedly affected. These results provide a reference for searching for neonicotinoid insecticides and agricultural fungicide candidates in the future.

## Figures and Tables

**Table 1:** Insecticidal activities of compounds **3a**–**3j**, **4a** and **5a**–**5i** and imidacloprid (IMI) against *T. Cinnabarinus*.

Compd.	Mortality (%) ^a,b^					
	250 mg/L	100 mg/L	50 mg/L	10 mg/L	1 mg/L	LC_50_ (mM) ^c^
**3a**	63	37	30	23	10	0.473
**3b**	63	37	23	13	7	0.523
**3c**	100	60	53	37	17	0.057
**3d**	100	97	93	57	30	0.011
**3e**	60	50	40	27	10	0.282
**3f**	90	58	45	21	8	0.122
**3g**	97	87	43	17	10	0.076
**3h**	90	50	33	23	17	0.159
**3i**	97	70	63	50	20	0.033
**3j**	93	80	37	27	7	0.079
**4a**	90	40	27	17	7	0.204
**5a**	87	60	47	30	13	0.096
**5b**	90	53	43	20	13	0.132
**5c**	100	67	57	43	33	0.025
**5d**	80	43	40	23	20	0.191
**5e**	97	80	60	20	12	0.064
**5f**	80	57	53	30	10	0.107
**5g**	70	30	20	17	3	0.404
**5h**	100	33	23	20	13	0.184
**5i**	90	47	30	23	10	0.173
**IMI**	90	60	33	30	27	0.135

**^a^** Temperature: 25 ± 2 °C; room humidity (RH): 65–80%; photoperiod: light/dark = 12/12 h. **^b^** Experimental size: 10 insects per group, three groups. **^c^** LC_50_ calculations were determined by Probit analysis using a maximum quasi-likelihood curve fitting algorithm.

**Table 2:** Insecticidal activities of compounds **3c**, **3d**, **3i**, **5c**, **5e** and imidacloprid (IMI) against *M. persicae*.

Compd.	Mortality (%) ^a,b^								
	24 h				48 h				
	50 mg/L	10 mg/L	1 mg/L	0.1 mg/L	50 mg/L	10 mg/L	1 mg/L	0.1 mg/L	LC_50_ (mM) ^c^
**3c**	63	39	30	17	87	43	37	30	0.0077
**3d**	53	27	17	13	93	63	40	33	0.0033
**3i**	57	30	20	10	90	83	70	37	0.0007
**5c**	40	33	20	10	70	50	40	27	0.0111
**5e**	40	33	20	7	87	57	43	23	0.0053
**IMI**	40	23	6	3	100	87	70	37	0.0010

**^a^** Temperature: 25 ± 2 °C; RH: 65–80%; photoperiod: light/dark = 12/12 h. **^b^** Experimental size: 10 insects per group, three groups. **^c^** LC_50_ calculations were determined by Probit analysis using a maximum quasi-likelihood curve fitting algorithm.

**Table 3:** Insecticidal activities of compounds **3c**, **3d**, **3i**, **5c**, **5e** and imidacloprid (IMI) against *B. brassicae*.

Compd.	Mortality (%) ^a,b^								
	24 h				48 h				
	50 mg/L	10 mg/L	1 mg/L	0.1 mg/L	50 mg/L	10 mg/L	1 mg/L	0.1 mg/L	LC_50_ (mM) ^c^
**3c**	37	30	20	13	70	63	40	20	0.0087
**3d**	67	40	33	17	90	70	60	33	0.0015
**3i**	60	43	30	17	83	67	57	27	0.0025
**5c**	30	20	13	7	60	50	33	13	0.0291
**5e**	53	37	23	13	67	57	37	23	0.0125
**IMI**	93	77	43	10	97	90	77	43	0.0006

**^a^** Temperature: 25 ± 2 °C; RH: 65–80%; photoperiod: light/dark = 12/12 h. **^b^** Experimental size: 10 insects per group, three groups. **^c^** LC_50_ calculations were determined by Probit analysis using a maximum quasi-likelihood curve fitting algorithm.

**Table 4:** Antifungal activities of compounds **3a**–**j**, **4**, **5a**–**i** and azoxystrobin against *F. oxysporum*, *M. oryzae*, *S. sclerotiorum*, *B. cinerea* at 50 mg/L.

Compd.	Inhibition (%)			
	*F. oxysporum*	*M. oryzae*	*S. sclerotiorum*	*B. cinerea*
**3a**	5.00	8.75	0.00	6.25
**3b**	23.33	12.50	7.08	5.00
**3c**	17.92	4.17	24.17	11.25
**3d**	5.42	11.67	0.00	0.00
**3e**	0.00	3.75	0.00	0.00
**3f**	11.67	11.25	6.25	0.00
**3g**	7.87	10.56	7.32	0.00
**3h**	0.00	0.00	8.33	0.00
**3i**	12.45	4.56	7.52	6.27
**3j**	5.42	10.83	10.83	6.25
**4**	45.00	21.67	65.83	18.33
**5a**	0.00	6.45	0.00	7.78
**5b**	5.42	0.00	0.00	0.00
**5c**	5.83	0.00	0.00	32.50
**5d**	0.00	9.58	11.67	0.00
**5e**	0.00	7.56	9.24	0.00
**5f**	2.92	16.25	6.25	0.00
**5g**	0.00	5.83	6.25	0.00
**5h**	0.00	9.58	10.83	0.00
**5i**	7.08	5.42	0.00	0.00
azoxystrobin	52.50	88.48	74.58	42.08

## Supplementary Materials

The representative compounds ^1^H and ^13^C NMR spectra are available online.
