# Supplementary material for: Facile Three-Component Synthesis, Insecticidal and Antifungal Evaluation of Novel Dihydropyridine Derivatives
Source: Molecules. 2018 Sep 21;23(10):2422. doi: 10.3390/molecules23102422 (PMC6222924; doi:10.3390/molecules23102422)
Supplement: Supplementary file 1 [file molecules-23-02422-s001.pdf]

Support information

Facile three-component synthesis, insecticidal and antifungal evaluation of novel Dihydropyridine derivatives

Guan-Zhou Yang[1†], Xiao-Fei Shang[1,2†], Pi-Le Cheng[1], Xiao-Dan Yin[1], Jia-Kai Zhu[1], Ying-Qian Liu[1*], Jing Zhang[3*], Zhi-Jun Zhang[1*]

[1] School of Pharmacy, Lanzhou University, Lanzhou 730000, People's Republic of China; yanggzh2016@lzu.edu.cn (G.Z.Y.); 1014153653@qq.com (P.L.C.); yinxd14@lzu.edu.cn (Y.X.D.); zhujk17@lzu.edu.cn (Z.J.K.).

[2] Lanzhou Institute of Husbandry and Pharmaceutical Sciences, Chinese Academy of Agricultural Sciences, Lanzhou 730000, People's Republic of China; shangxf928@126.com (X.F.S.)

[3] Environment and Plant Protection Institute, Chinese Academy of Tropical Agricultural Sciences, Haikou 571010, People's Republic of China

* Correspondence: yqliu@lzu.edu.cn (Y.Q.L.); zh-jing99@163.com (J.Z.); zhangzhijun198803@163.com (Z.J.Z.) Tel.: +86-898-66969260

† These authors contributed equally to this work.

Representative compounds ^{1}H and ^{13}C NMR spectra

Contents

Copies of ^{1}H and ^{13}C NMR Spectra for Compounds 3c 2

Copies of ^{1}H and ^{13}C NMR Spectra for Compounds 3d 3

Copies of ^{1}H and ^{13}C NMR Spectra for Compounds 3i 4

Copies of ^{1}H and ^{13}C NMR Spectra for Compounds 5c 5

Copies of ^{1}H and ^{13}C NMR Spectra for Compounds 5e 6

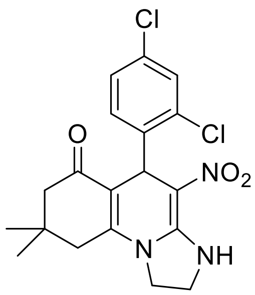

Cl
Cl
O
NO2
N
NH

3c

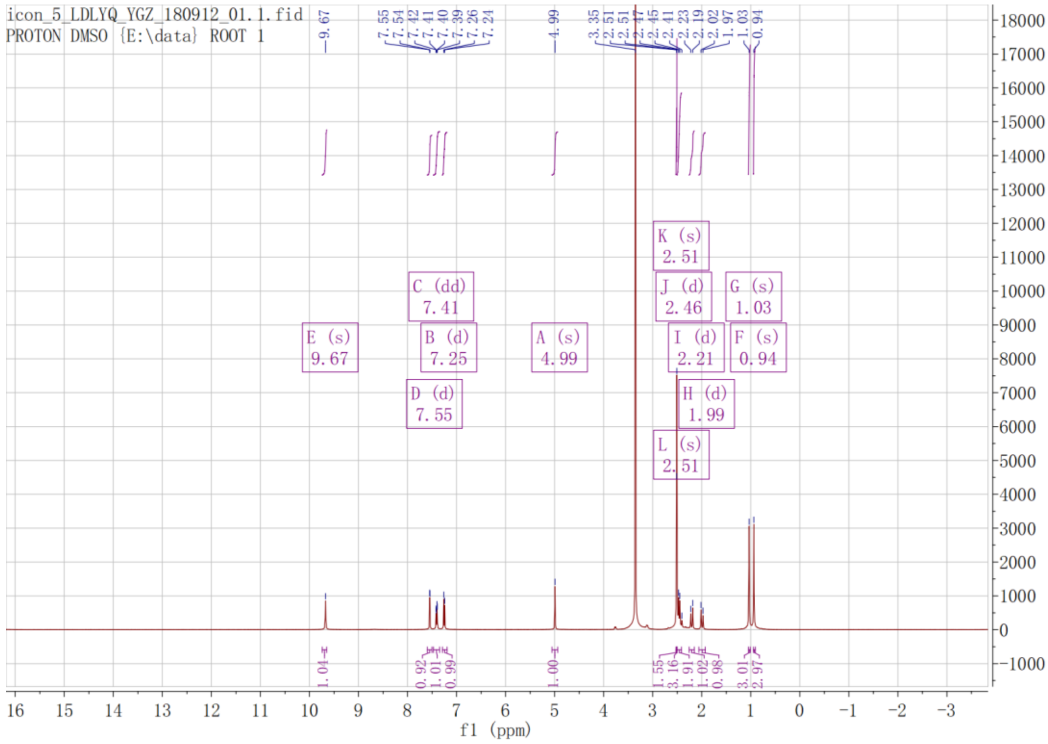

icon_5_LDLYQ_YGZ_180912_01.1.fid
PROTON DMSO {E:\data} ROOT 1
9.67
7.55 7.54 7.42 7.41 7.40 7.39 7.26 7.24
4.99
3.35 2.51 2.51 2.47 2.45 2.41 2.23 2.19 2.02 1.97 1.03 0.94
E (s) 9.67
C (dd) 7.41
B (d) 7.25
D (d) 7.55
A (s) 4.99
K (s) 2.51
J (d) 2.46
I (d) 2.21
H (d) 1.99
L (s) 2.51
G (s) 1.03
F (s) 0.94
1.04
0.92 1.01 0.99
1.00
1.55 3.16 1.91 1.02 0.98
3.01 2.97
f1 (ppm)

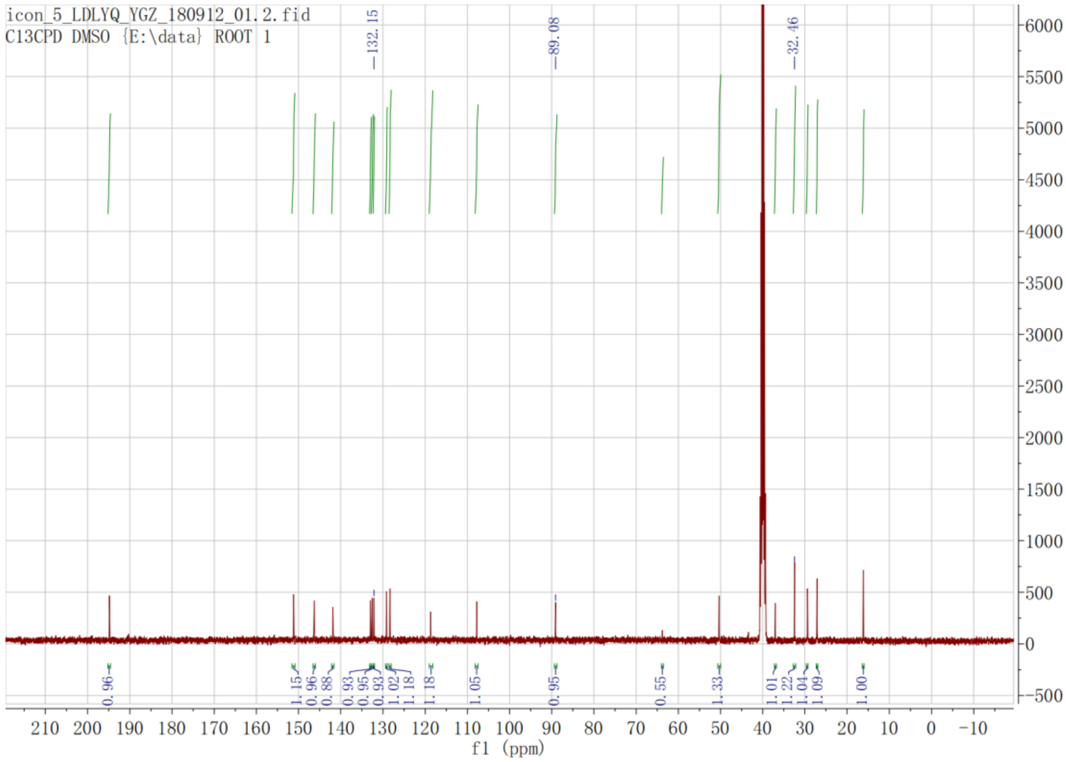

icon_5_LDLYQ_YGZ_180912_01.2.fid
C13CPD DMSO {E:\data} ROOT 1
132.15
89.08
32.46
0.96
1.15 0.96 0.88 0.93 0.95 0.93 1.02 1.18 1.05
0.95
0.55
1.33
1.01 1.22 1.04 1.09
1.00
f1 (ppm)

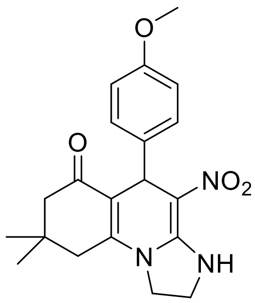
O
NO2
N
NH

3d

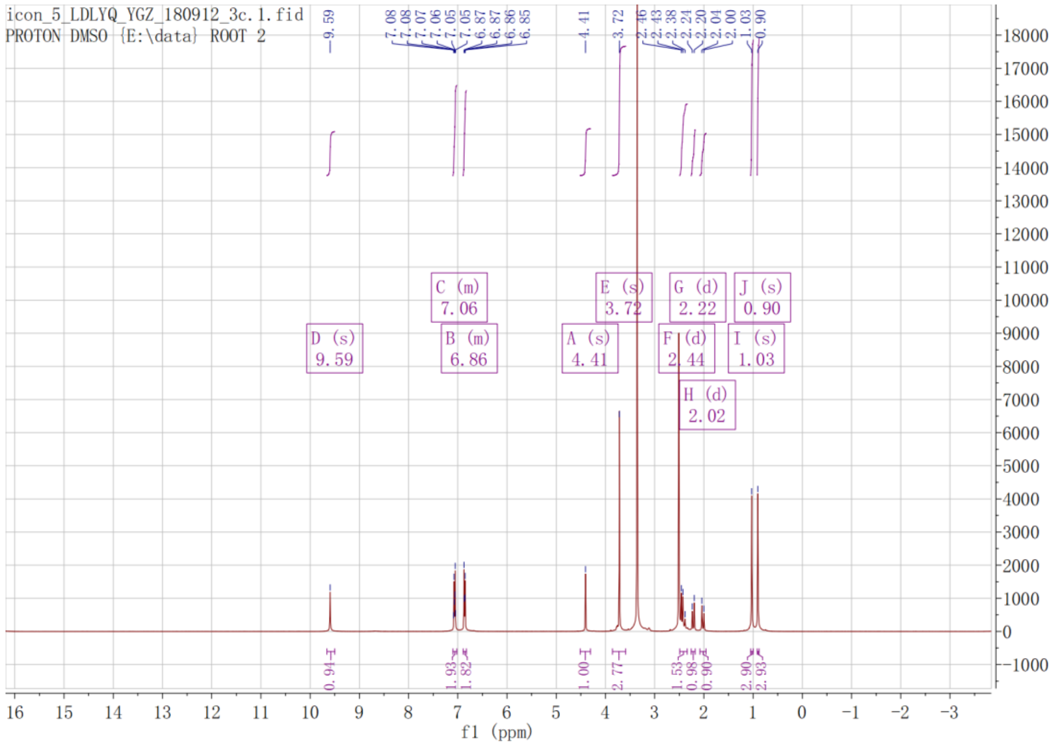
icon_5_LDLYQ_YGZ_180912_3c.1.fid
PROTON DMSO {E:\data} ROOT 2
D (s) 9.59
C (m) 7.06
B (m) 6.86
A (s) 4.41
E (s) 3.72
F (d) 2.44
G (d) 2.22
H (d) 2.02
I (s) 1.03
J (s) 0.90
f1 (ppm)

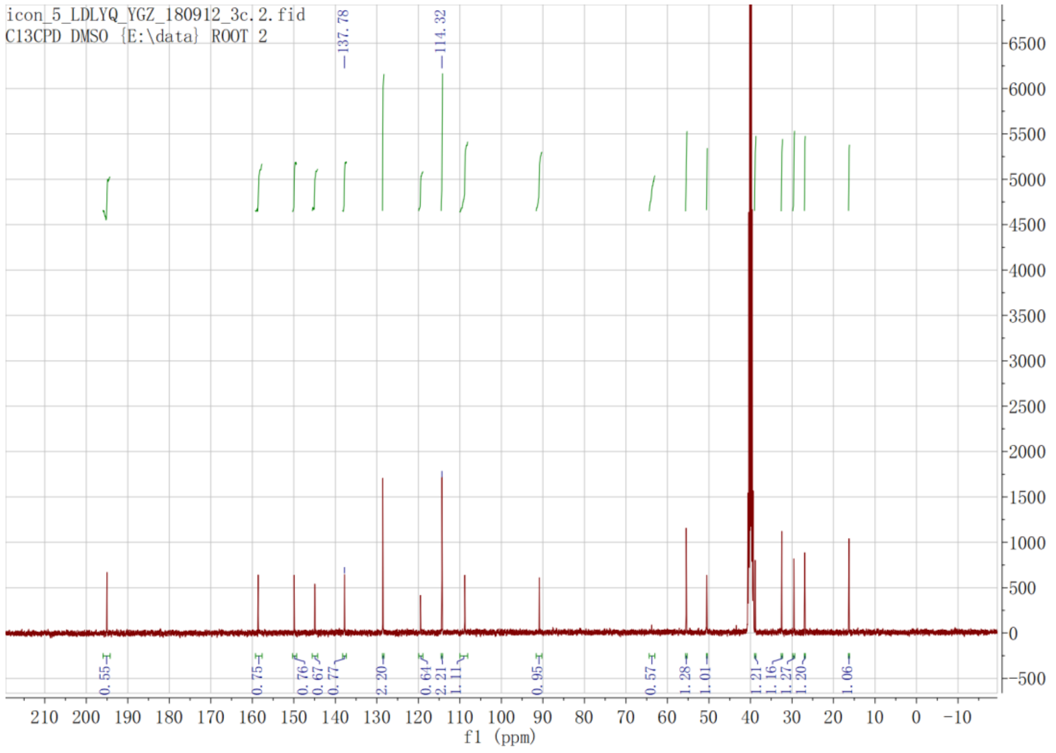
icon_5_LDLYQ_YGZ_180912_3c.2.fid
C13CPD DMSO {E:\data} ROOT 2
137.78
114.32
f1 (ppm)

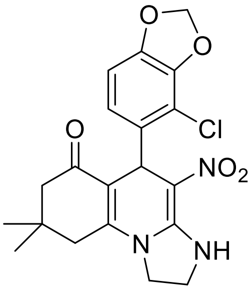
O
O
O
Cl
NO2
N
NH

3i

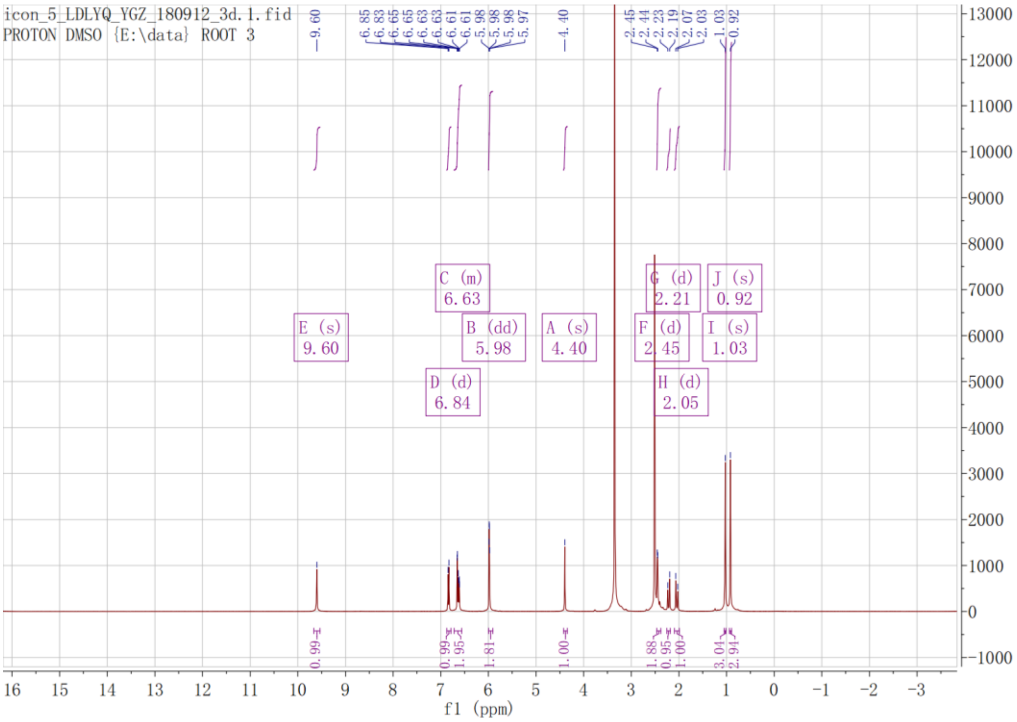
icon_5_LDLYQ_YGZ_180912_3d.1.fid
PROTON DMSO {E:\data} ROOT 3
9.60
6.85 6.83 6.65 6.65 6.63 6.63 6.61 6.61 5.98 5.98 5.98 5.97
4.40
2.45 2.44 2.23 2.19 2.07 2.03 1.03 0.92
E (s) 9.60
C (m) 6.63
D (d) 6.84
B (dd) 5.98
A (s) 4.40
G (d) 2.21
F (d) 2.45
H (d) 2.05
J (s) 0.92
I (s) 1.03
0.99 0.99 1.95 1.81 1.00 1.88 0.95 1.00 3.04 2.94
f1 (ppm)

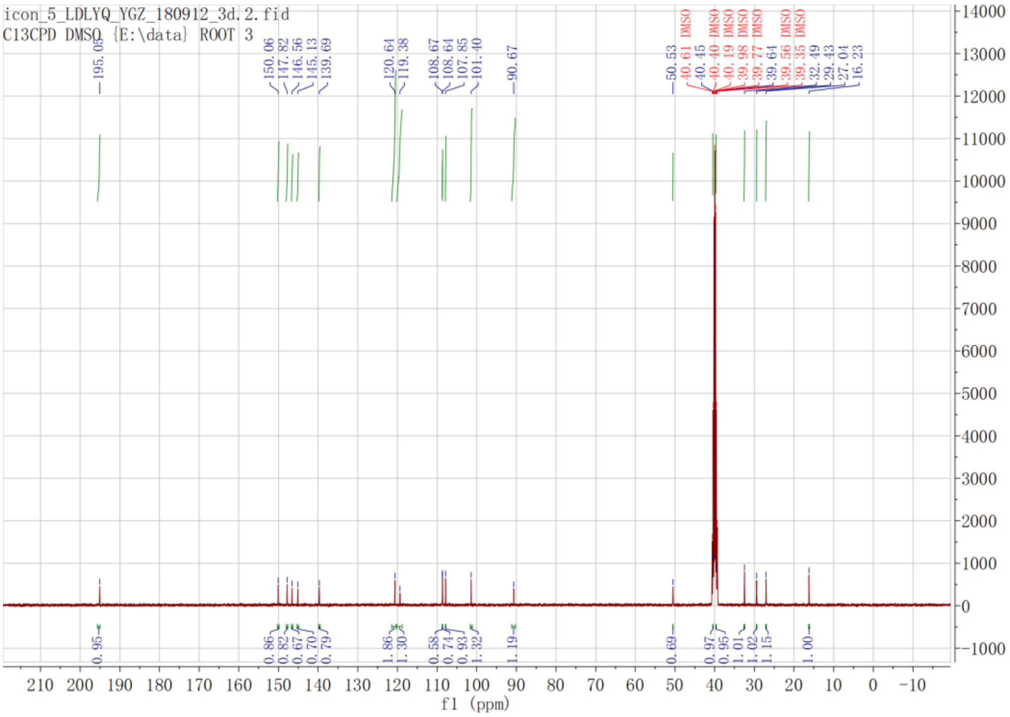
icon_5_LDLYQ_YGZ_180912_3d.2.fid
C13CPD DMSO {E:\data} ROOT 3
195.05
150.06 147.82 146.56 145.13 139.69
120.64 119.38
108.67 108.64 107.85 101.40
90.67
50.53
40.61 DMSO 40.45 40.40 DMSO 40.19 DMSO 39.98 DMSO 39.77 DMSO 39.64 39.56 DMSO 39.35 DMSO 32.49 29.43 27.04 16.23
0.95 0.86 0.82 0.67 0.70 0.79 1.86 1.30 0.58 0.74 0.93 1.32 1.19 0.69 0.97 0.95 1.01 1.02 1.15 1.00
f1 (ppm)

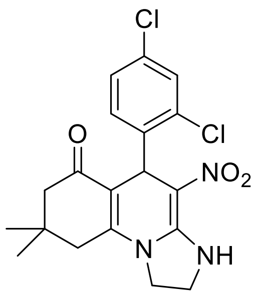

5c

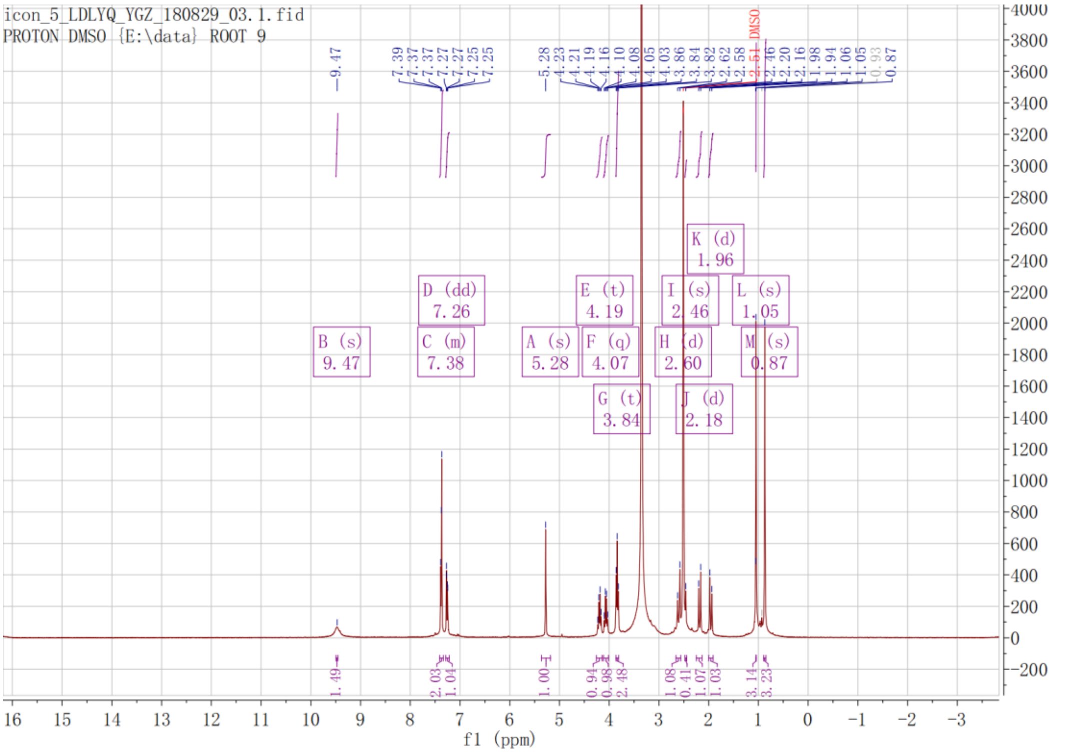

icon_5_LDLYQ_YGZ_180829_03.1.fid
PROTON DMSO {E:\data} ROOT 9
9.47
7.39
7.37
7.27
7.27
7.25
7.25
5.28
4.23
4.21
4.19
4.16
4.10
4.08
4.05
4.03
3.86
3.84
3.82
2.62
2.58
2.51 DMSO
2.46
2.20
2.16
1.98
1.94
1.06
1.05
0.93
0.87
B (s) 9.47
C (m) 7.38
D (dd) 7.26
A (s) 5.28
E (t) 4.19
F (q) 4.07
G (t) 3.84
H (d) 2.60
I (s) 2.46
J (d) 2.18
K (d) 1.96
L (s) 1.05
M (s) 0.87
1.49
2.03
1.04
1.00
0.94
0.98
2.48
1.08
0.41
1.07
1.03
3.14
3.23
f1 (ppm)

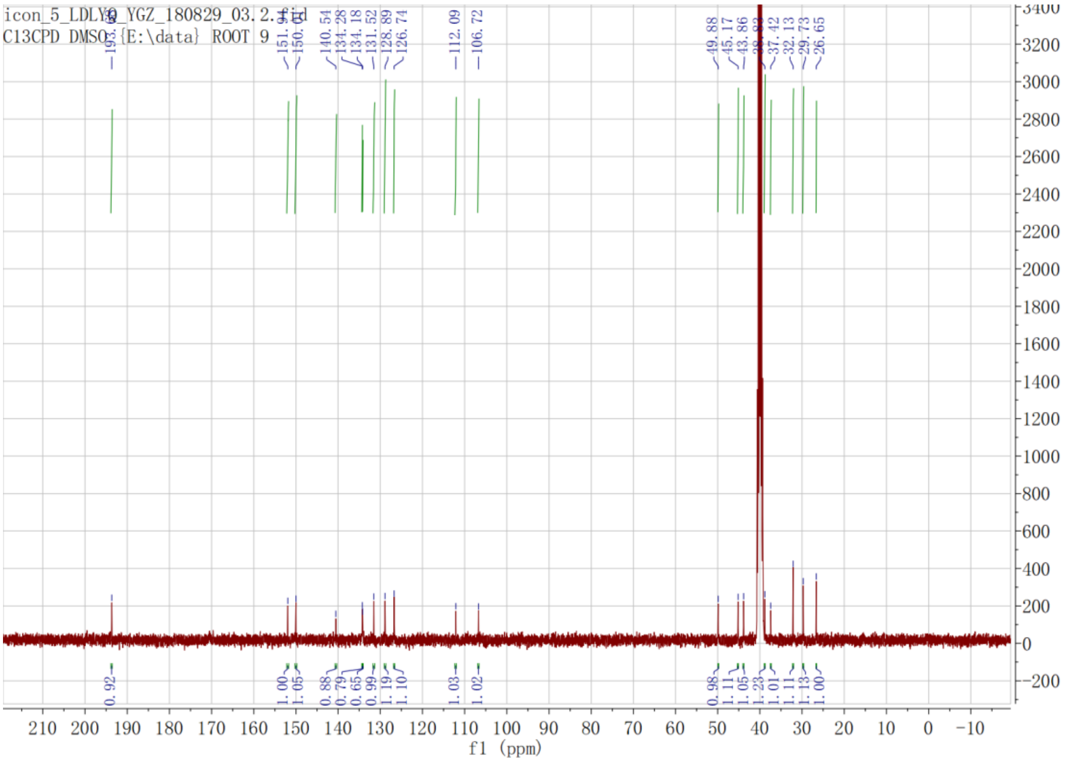

icon_5_LDLYQ_YGZ_180829_03.2.fid
C13CPD DMSO {E:\data} ROOT 9
140.54
134.28
134.18
131.52
128.89
126.74
112.09
106.72
45.17
43.86
37.42
32.13
29.73
26.65
0.92
1.00
1.05
0.88
0.79
0.65
0.99
1.19
1.10
1.03
1.02
0.98
1.11
1.05
1.23
1.01
1.11
1.13
1.00
f1 (ppm)

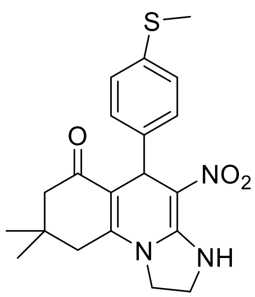
S
O
NO2
N
NH

5e

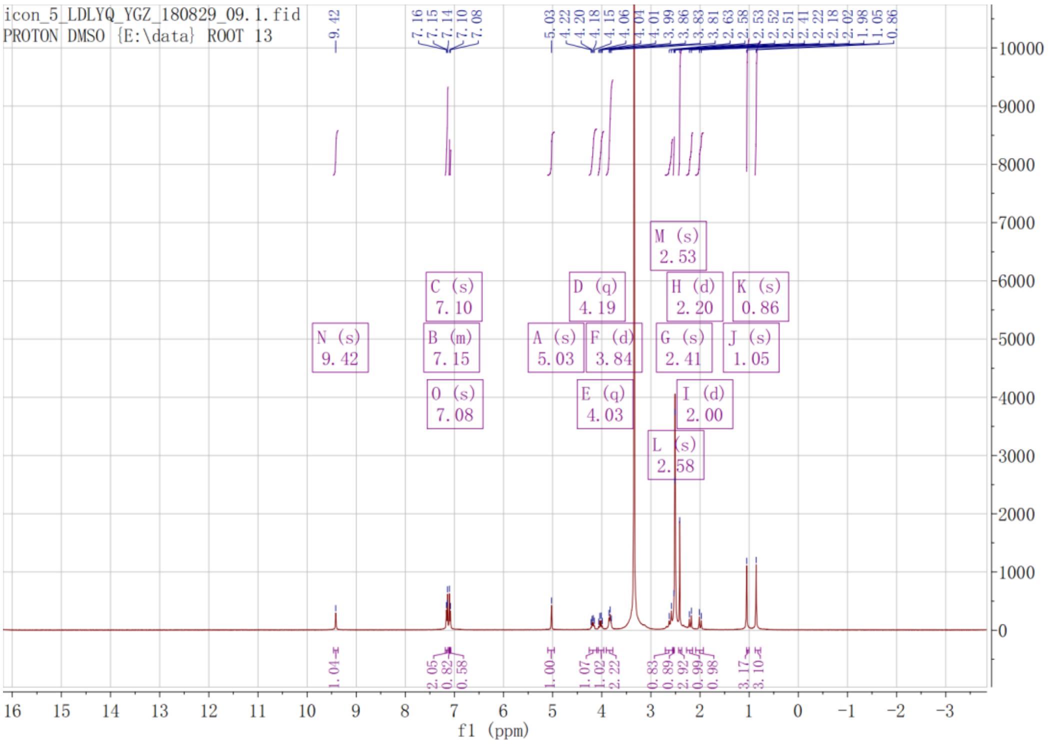
icon_5_LDLYQ_YGZ_180829_09.1.fid
PROTON DMSO {E:\data} ROOT 13
f1 (ppm)

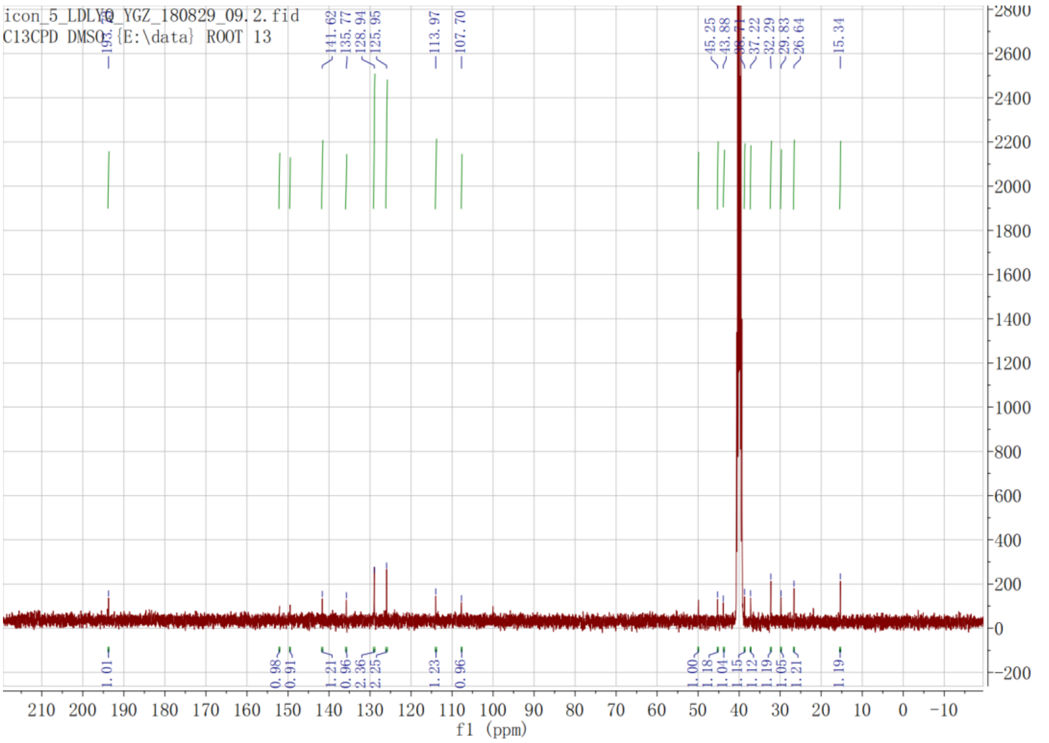
icon_5_LDLYQ_YGZ_180829_09.2.fid
C13CPD DMSO {E:\data} ROOT 13
f1 (ppm)
